# A Lightweight RGB Vision-Based Express Parcel Detection Framework with Active Learning and Quantization-Aware Training

**DOI:** 10.3390/s26196036

**Published:** 2026-09-23

**Authors:** An-Qi Wu, Fan Guo, Weijun Yang, Rui-Feng Wang, Pingfan Hu

**Affiliations:** 1 Yantai Institute, China Agricultural University, Yantai 264670, China; anqi.wu@uga.edu (A.-Q.W.); yangwj@cau.edu.cn (W.Y.); 2 Department of Crop and Soil Sciences, College of Agriculture and Environmental Sciences, University of Georgia, Tifton, GA 31793, USA; 3 Sichuan Energy Internet Research Institute, Tsinghua University, Chengdu 610213, China; guofan@tsinghua-eiri.org; 4 Artie McFerrin Department of Chemical Engineering, Texas A&M University, College Station, TX 77843, USA

**Keywords:** express parcel detection, lightweight object detection, active learning, YOLO, quantization-aware training, intelligent logistics

## Abstract

This study presents a lightweight RGB vision-based express-parcel detection framework for complex logistics environments. An Express Parcel Recognition (EPR) dataset is first constructed from RGB images acquired through multi-source data collection, followed by unified re-annotation and Active Learning-based pseudo-labeling to reduce manual annotation effort while maintaining label consistency. Based on YOLOv11s, a lightweight detector termed EPR-YOLO is developed by integrating SFAConv and NGAM to strengthen multi-scale feature extraction and global semantic modeling from RGB visual inputs while controlling computational complexity. Quantization-Aware Training is further introduced to obtain an INT8-quantized model with reduced storage requirements and limited accuracy degradation, providing a basis for subsequent lightweight deployment. Comparative experiments, ablation studies, and independent test set evaluations demonstrate that EPR-YOLO achieves an mAP_50_ of 71.654%, improving the YOLOv11s baseline by 0.854 percentage points while reducing the parameter count by 9.5%; the quantized model further retains most of the detection capability of its floating-point counterpart. In addition, a FastAPI-based Web application is implemented to support RGB image acquisition-based single-image and batch detection, result visualization, and online inference. The proposed framework therefore provides an integrated sensing-to-deployment solution covering RGB image-based parcel perception, dataset construction, lightweight model optimization, quantized deployment, and application-level validation for intelligent logistics systems.

## 1. Introduction

In recent years, the rapid expansion of e-commerce and modern logistics systems has substantially increased parcel-handling volumes, placing greater demands on visual perception for automated sorting, intelligent warehousing, and robotic manipulation [1]. Accurate detection and localization of express parcels are fundamental to downstream operations such as sorting, grasping, and material transfer [2]. However, unlike structured industrial vision environments, real-world logistics scenes exhibit substantial variability in parcel size, shape, packaging material, and surface condition, while frequently involving damage, deformation, occlusion, stacking, and irregular placement [3]. Variations in background complexity, illumination, and viewing angle further complicate reliable object recognition and localization [4,5]. Consequently, developing parcel detection methods that jointly achieve high accuracy, robustness, and real-time performance is an important technical requirement for automated perception in intelligent logistics systems [6].

Early parcel recognition systems primarily relied on manual sorting, barcode and shipping-label parsing, optical character recognition, and conventional machine-vision methods based on handcrafted features [7,8]. Although these approaches can perform adequately under relatively controlled imaging conditions, their robustness may be affected by occlusion, contamination, deformation, irregular placement, and complex backgrounds. With the development of deep learning, convolutional neural network-based object detectors have increasingly been introduced into logistics and parcel-handling applications. Existing studies have investigated parcel localization using Faster R-CNN [4], lightweight deep-learning models for logistics parcel detection [6], and CNN-based parcel detection and tracking on conveyor belts [9]. Related research has also explored artificial intelligence for postal and parcel sorting [10] and deep learning-based recognition of product packaging [11], further demonstrating the potential of visual perception for automated logistics operations. Among contemporary detection architectures, YOLO-based models have attracted particular attention because of their end-to-end detection pipeline and favorable balance between detection performance and computational efficiency [12,13]. These developments provide an important technical foundation for real-time parcel perception in automated sorting, warehousing, and downstream robotic operations.

Despite these advances, parcel detection in practical logistics environments remains limited by several challenges [9]. First, public parcel datasets are fragmented and often use inconsistent category definitions and annotation protocols, while fully manual construction of large-scale, high-quality datasets is labor-intensive [14]. Second, scale variation, localized damage, ambiguous boundaries, occlusion, and background interference require stronger multi-scale feature extraction and global semantic modeling [15], yet simply increasing model capacity raises parameter count and computational cost [13]. Third, practical logistics applications require detectors to balance recognition performance with computational efficiency and low-precision inference capability [16]. Although previous studies have demonstrated the feasibility of deep learning for parcel localization, sorting, tracking, and packaging recognition [4,6,9,10,11], dataset construction, efficient model optimization for diverse parcel conditions, and low-precision model optimization have often been investigated separately. Consequently, an integrated framework that jointly addresses dataset construction, lightweight parcel detection, quantization, and application-level validation remains desirable.

To address these limitations, this study develops a lightweight express-parcel detection framework with the following objectives: (1) to construct the Express Parcel Recognition (EPR) dataset by integrating open-source, Internet-sourced, and field-acquired images with unified re-annotation and Active Learning-based iterative pseudo-labeling; (2) to develop EPR-YOLO based on YOLOv11s by incorporating Selective Feature Aggregation Convolution (SFAConv) and the NeLU-enhanced Global Attention Mechanism (NGAM) for efficient multi-scale feature extraction and global semantic modeling; (3) to apply Quantization-Aware Training (QAT) for INT8 deployment and evaluate performance retention on an independent test set; and (4) to implement a FastAPI-based Web application for online inference, visualization, and system-level deployment validation. Together, these objectives establish an integrated pipeline from dataset construction and model optimization to quantized deployment and application validation.

## 2. Materials and Methods

### 2.1. Dataset Construction

#### 2.1.1. Data Source

To satisfy the requirements of intelligent express-parcel detection for data-source diversity, representative sample distributions, and broad scenario coverage, this study adopted a multi-source heterogeneous data acquisition strategy. Raw images were collected from three sources: public datasets, Internet-crawled images, and field photography, yielding a total of 12,798 images. Of these, approximately 56.8% (7269 images) were obtained from public datasets, 37.3% (4774 images) from Internet crawling, and 5.9% (755 images) from field acquisition. These sources are complementary in terms of imaging environments, parcel appearance, background complexity, and image quality, thereby improving the coverage of the raw dataset across practical express-logistics scenarios.

Public datasets constituted the primary source of raw images. During data collection, relevant datasets were systematically retrieved from public platforms such as Roboflow and Kaggle, with priority given to datasets from reliable sources that provided adequate sample sizes, acceptable image quality, and strong relevance to express-parcel detection. However, substantial inconsistencies were observed across datasets in class taxonomy, label naming, definitions of damage status, and annotation granularity, with some labels exhibiting semantic overlap or inconsistent definitions. Directly merging the original annotations could therefore introduce ambiguous class boundaries and inconsistent labeling standards, ultimately compromising model training. Accordingly, the original labels provided by these datasets were not directly retained. Instead, only images meeting the requirements of this study were preserved and subsequently re-annotated according to a unified class taxonomy during data preprocessing.

To increase samples for specific parcel categories and condition states, a web-crawling program [17,18] was used to acquire images of boxes, envelopes, poly mailers, tubes, irregular parcels, and their damaged variants. Given the heterogeneous quality of Internet-sourced data, low-resolution, blurred, distorted, duplicated, visually ambiguous, and category-irrelevant images were removed to reduce annotation noise and improve data reliability [19]. Field photography was further conducted at the Yantai Institute of China Agricultural University (Yantai, Shandong, China; 37.4344° N, 121.5213° E) to supplement online data with samples collected under real logistics conditions. The acquired images covered variations in parcel arrangement, imaging distance, background complexity, and parcel condition, thereby improving the environmental diversity and practical representativeness of the raw dataset.

#### 2.1.2. Pseudo-Label Iteration Dataset Construction

Building on the multi-source raw image collection, we further constructed the express-parcel recognition dataset through a combination of standardized preprocessing, unified re-annotation, and Active Learning-based pseudo-label iteration, as illustrated in Figure 1. First, the raw images underwent quality control to remove blurred, improperly exposed, duplicated, and task-irrelevant samples, after which all retained images were converted to JPG format. To eliminate annotation inconsistencies across data sources and ensure a unified class definition, all original labels were discarded and the valid images were re-annotated according to the eight-class parcel taxonomy defined in this study: box, damaged envelope, envelope, poly mailer, damaged poly mailer, damaged box, tube, and irregular. After image screening and standardization, 5603 valid images were retained and designated as the Raw Set, which served as the basis for subsequent annotation, Active Learning iteration, and detector development [20].

To improve large-scale annotation efficiency while maintaining label reliability, an Active Learning-based pseudo-labeling strategy was then employed [21,22]. Pseudo-labeling and subsequent label refinement have been increasingly investigated as effective approaches for exploiting unlabeled or partially labeled data while reducing dependence on exhaustive manual annotation. Recent studies have further demonstrated the utility of explicit pseudo-label correction for improving learning from automatically generated annotations, particularly for samples with different levels of recognition difficulty [23]. To identify a suitable baseline detector for the subsequent iterative labeling process, the 5603 images in the Raw Set were randomly divided into seven subsets, denoted Raw-Subset 1–7. Raw-Subset 1–6 each contained 800 images, whereas Raw-Subset 7 contained 803 images. The 800 images in Raw-Subset 1 were manually annotated using Make Sense AI (https://www.makesense.ai/, accessed on 1 September 2026) to form Ground Truth (GT) Subset 1, which was used as the initial training set for baseline model selection. Specifically, 12 lightweight and small-scale models from the YOLOv8–YOLOv12 [24,25,26,27,28] and YOLO26 [29] series were trained on GT Subset 1. To reduce the influence of random initialization and other stochastic factors, each model was trained using three different random seeds, resulting in 36 independent experiments. Model performance was comprehensively evaluated using Precision (*P*), Recall (*R*), mAP_50_, mAP, and $F_{1}$ Score. As shown in Table 1, although individual models exhibited advantages on specific metrics, YOLOv11s achieved the highest mAP while maintaining a favorable balance in Precision and $F_{1}$ Score. YOLOv11s was therefore selected as the baseline detector for the subsequent Active Learning-based pseudo-labeling iterations.

After baseline selection using GT Subset 1, the remaining 6 Raw-Subsets were sequentially incorporated through iterative pseudo-label generation and manual correction. In each iteration, the selected YOLOv11s model was applied to an unlabeled Raw-Subset of approximately 800 images to generate pseudo-labels, which were subsequently inspected and corrected for classification errors, inaccurate localization, missed detections, and false positives. The corrected subset was then merged with the existing annotated data to form an expanded cumulative training set. YOLOv11s was retrained on each updated dataset using three random seeds, and the best-performing model was used for pseudo-label generation in the subsequent iteration. This procedure progressively expanded the labeled dataset while incorporating increasingly diverse parcel samples and logistics scenarios. As shown in Figure 2, mAP and $F_{1}$ generally increased as the labeled sample size grew across iterations, indicating improved detection performance with progressive dataset expansion.

Following the Active Learning-based pseudo-labeling iterations described above, the Express Parcel Recognition (EPR) dataset was established, with representative samples shown in Figure 3. The final dataset comprises 5603 annotated images containing 8274 object instances across eight common express-parcel categories and their damaged variants. This dataset provides a consistent data foundation for subsequent model benchmarking, training of the proposed model, ablation studies, and performance evaluation.

### 2.2. Baseline Selection

For express-parcel detection, the baseline model should provide not only high detection accuracy but also favorable computational efficiency and deployment feasibility to support subsequent Web application development and lightweight deployment. As a representative family of one-stage object detectors, You Only Look Once (YOLO) models have been widely adopted across diverse detection tasks because of their streamlined architectures, fast inference, and deployment efficiency [30,31,32,33]. Recent advances have produced multiple generations, including YOLOv8 [24], YOLOv9 [25,34], YOLOv10 [26], YOLOv11 [17,27], YOLOv12 [28], and YOLO26 [29]. These models offer competitive trade-offs among detection accuracy, inference efficiency, and deployment adaptability, making them representative candidates for baseline selection in express-parcel detection. Deep-learning-based real-time visual perception has also been investigated in logistics-vehicle monitoring and in visually complex field applications involving crop and fruit detection, tracking, phenotyping, and robot perception [35,36,37,38,39,40,41,42]. These studies provide broader methodological context for evaluating detector architectures under substantial variation in target scale, appearance, and operating conditions.

To identify a suitable baseline detector for subsequent architectural improvement and model optimization, lightweight and small-scale variants from the YOLOv8, YOLOv9, YOLOv10, YOLOv11, YOLOv12, and YOLO26 families were comparatively evaluated on the complete EPR dataset. Specifically, the dataset was divided into training and validation sets at an 8:2 ratio. This split was used only for preliminary baseline selection, where the objective was to identify a suitable baseline architecture rather than to conduct final generalization evaluation. Therefore, no independent test set was reserved at this stage, allowing more samples to be used for model training and validation. To reduce the dependence of the results on a single random split, three independent dataset partitions were generated using different random seeds, and the corresponding lightweight and small-scale models were trained separately under each split. Detection performance was evaluated using *P*, *R*, mAP_50_, mAP, and $F_{1}$, while computational complexity and model size were quantified by GFLOPs and parameter count (Params), respectively. This protocol enabled a joint comparison of predictive performance and deployment cost across candidate models.

As shown in Table 2, the s variants generally outperformed their corresponding n/t counterparts in Recall, mAP, and $F_{1}$, indicating that increased model capacity can improve feature representation and overall detection performance. This improvement, however, was accompanied by noticeably higher GFLOPs and parameter counts, highlighting the inherent trade-off between detection performance and computational cost. Baseline selection should therefore not rely on a single accuracy metric, but instead consider predictive performance, model complexity, and training stability jointly.

Among all evaluated candidates, YOLOv11s achieved the strongest overall detection performance, with the highest Recall (73.072%), mAP_50_ (72.454%), mAP (67.916%), and $F_{1}$ (80.088%). Its Precision was only marginally lower than that of YOLO26n (88.597% vs. 89.059%). Meanwhile, YOLOv11s required 21.6 GFLOPs and contained 9.43 M parameters, values comparable to the 21.5 GFLOPs and 9.25 M parameters of YOLOv12s. This suggests that the performance advantage of YOLOv11s was not primarily achieved through additional computational overhead, but rather through more effective detection at a similar level of model complexity. Compared with the YOLO26 variants, YOLOv11s exhibited slightly lower Precision but clear advantages in Recall, mAP_50_, mAP, and $F_{1}$, indicating a more balanced detection capability with fewer missed targets.

Moreover, YOLOv11s exhibited consistently low standard deviations across the three independent random experiments, demonstrating robustness to variations in dataset partitioning and good training reproducibility. Considering detection accuracy, recall capability, model complexity, and training stability together, YOLOv11s was ultimately selected as the baseline detector for subsequent pseudo-label generation, architectural refinement, and model optimization.

### 2.3. Model Optimization

#### 2.3.1. Selective Feature Aggregation Convolution

To reduce computational complexity and improve detection efficiency, this study introduces Selective Multi-Scale Feature Aggregation Convolution (SFAConv) [43], whose architecture is illustrated in Figure 4. SFAConv adopts a compact hierarchical feature-processing scheme. Specifically, a 1×1 pointwise convolution is first applied to compress the input channels and, when required, simultaneously perform spatial downsampling. This operation produces a more compact intermediate feature representation, thereby reducing the parameter count and computational cost of the subsequent multi-scale spatial feature extraction process. Multi-scale representation has been widely investigated in object detection because targets with substantial variations in spatial extent may require feature responses at different receptive-field scales [15]. Related studies have also explored adaptive cross-channel dependencies and multi-domain feature fusion to strengthen object representation under complex backgrounds and occlusion conditions [44,45].

After channel compression, SFAConv employs three parallel depthwise separable convolution branches to extract spatial features at different scales, with effective receptive fields approximating 3×3, 5×5, and 7×7, respectively. The larger receptive fields are implemented using dilated convolutions, enabling an expanded spatial modeling range without substantially increasing the parameter count or computational cost. The three branches are designed to capture fine-grained local textures, intermediate-scale morphological characteristics, and broader structural patterns, respectively, allowing the module to jointly represent express parcels with varying sizes and appearance conditions.

Rather than fusing multi-scale features using fixed weights, SFAConv further incorporates a Selective Kernel (SK) gating mechanism to adaptively reweight the outputs of the three parallel branches. First, the output features from the individual scale branches are denoted as:

(1) $$B=\left\{b_{1},b_{2},b_{3}\right\},\;b_{i}\in R^{B\times m\times H^{\prime }\times W^{\prime }}$$

Subsequently, the mean of the three branch features is subjected to global average pooling, followed by channel compression, nonlinear transformation, and channel expansion to generate the scale-selection weights:

(2) $$w=\mathrm{Softmax}\left(f_{\mathrm{fc}↑}\left(\sigma \left(f_{\mathrm{fc}↓}\left(\mathrm{GAP}(\frac{1}{3}\sum_{i=1}^{3}b_{i})\right)\right)\right)\right)$$

Finally, the outputs of the individual scale branches are adaptively fused according to the learned weights:

(3) $$x=\sum_{i=1}^{3}w_{i}⊙b_{i}$$

Here, GAP(·) denotes global average pooling; $f_{\mathrm{fc}↓}$ and $f_{\mathrm{fc}↑}$ denote channel compression and channel expansion, respectively, both implemented using 1×1 convolutions; σ(·) denotes the nonlinear activation function; and ⊙ represents channel-wise weighting. Through this selective fusion mechanism, the model can dynamically adjust the responses of different scale branches according to the input feature content and imaging conditions, thereby emphasizing more discriminative scale-specific information and improving multi-scale representation for targets with substantial size variation, localized damage, and complex backgrounds.

After multi-scale feature fusion, SFAConv employs a 1×1 pointwise convolution to restore the feature channels to $C_{\mathrm{out}}$, followed by an Efficient Channel Attention (ECA) module [46] for lightweight channel recalibration. ECA models local cross-channel dependencies with minimal additional computational overhead, strengthening channel responses relevant to target recognition while suppressing redundant information. The overall computation of SFAConv can be formulated as:

(4) $$Y=F_{\mathrm{ECA}}\left(f_{1\times 1}^{↑}\left(\sum_{i=1}^{3}w_{i}⊙f_{\mathrm{DW}}^{\left(i\right)}\left(f_{1\times 1}^{↓}\left(X\right)\right)\right)\right)+1_{\left\{s=1,C_{m}=C_{\mathrm{out}}\right\}}\cdot X$$

Here, $f_{1\times 1}^{↓}\left(X\right)$ and $f_{1\times 1}^{↑}$ denote channel reduction and channel expansion implemented by pointwise convolutions, respectively; $f_{\mathrm{DW}}^{\left(i\right)}$ denotes the $i^{th}$ depthwise convolution branch; $F_{\mathrm{ECA}}$ represents the ECA-based channel recalibration function; and the indicator function $1_{\left\{s=1,\;C_{m}=C_{\mathrm{out}}\right\}}$ determines whether the residual connection condition is satisfied. When the spatial resolution remains unchanged and the input and output channel dimensions are compatible, a residual connection is applied to preserve the original feature information. This design mitigates potential information loss introduced by lightweight feature transformations and facilitates gradient propagation.

Overall, SFAConv is designed to preserve strong feature representation while controlling model complexity through channel compression, parallel multi-scale depthwise convolutions, adaptive scale selection, and lightweight channel recalibration. Within the YOLO framework, selected 3×3 convolutional layers in the Backbone are replaced with SFAConv without altering the overall hierarchical structure of the network, enabling more efficient multi-scale feature extraction. This design allows the detector to jointly capture parcel information at different spatial scales, including local textures, global contours, and damaged regions, thereby achieving a more favorable balance between detection accuracy and computational efficiency and providing an effective architectural basis for real-time detection of express parcels with varying categories and conditions in complex logistics environments.

#### 2.3.2. NeLU-Enhanced Global Attention Mechanism

Express parcels in complex logistics environments are often characterized by substantial scale variation, subtle localized damage, frequent occlusion, and strong background interference. Accordingly, an effective detector must not only preserve discriminative local details but also model cross-channel dependencies and global spatial context. To this end, this study introduces a NeLU-enhanced Global Attention Mechanism (NGAM) module [47] into the deeper stages of the YOLOv11s Backbone to strengthen global semantic modeling of high-level features.

Attention mechanisms have also been investigated across different visual and signal-analysis tasks to selectively emphasize informative representations and suppress task-irrelevant responses [48]. In object detection, such adaptive feature weighting is particularly relevant when targets are affected by occlusion, subtle local differences, and background interference. NGAM is derived from the Global Attention Mechanism (GAM) [49] by introducing the Negative-slope Linear Unit (NeLU) activation function [50]. GAM combines channel and spatial attention to model inter-channel dependencies and spatial contextual relationships, thereby enhancing target-relevant semantic responses while suppressing background interference. However, its nonlinear transformations commonly rely on Sigmoid or ReLU, which may suffer from gradient saturation or inactive responses for negative inputs. To improve nonlinear feature transformation and information propagation in deep representations, NGAM replaces these activations with NeLU. Its mathematical definition and derivative are given as follows:

(5) $$\mathrm{NeLU}\left(x\right)=\left\{\begin{array}{cc} x, & x>0\\ -\frac{\alpha }{1+x^{2}}, & x\leq 0 \end{array}\right.$$

(6) $$\frac{d}{dx}\mathrm{NeLU}\left(x\right)=\left\{\begin{array}{cc} 1, & x>0\\ \frac{2\alpha x}{{\left(1+x^{2}\right)}^{2}}, & x\leq 0 \end{array}\right.$$

Here, α=0.2 controls the negative-response magnitude of NeLU. For x>0, NeLU retains the linear response of ReLU, whereas for x≤0, it preserves negative-input information through a smooth nonlinear response, reducing neuron inactivity and gradient attenuation. Compared with Sigmoid, NeLU also avoids exponential computation while providing flexible nonlinear transformation. Based on these properties, NGAM introduces NeLU into both the channel- and spatial-attention branches of GAM and is embedded in the deeper stages of the YOLOv11s Backbone, where larger receptive fields and richer semantic features enable stronger global dependency modeling across channels and spatial locations. This design improves the representation of occluded parcels, complex backgrounds, and varying damage conditions. The detailed architecture of NGAM is illustrated in Figure 5.

#### 2.3.3. Quantization Aware Training

Quantization-Aware Training (QAT), first introduced by Google in 2018 [51], explicitly simulates low-precision quantization during training so that the model can adapt to the numerical errors introduced when floating-point computations are converted to integer arithmetic. Specifically, QAT inserts fake-quantization operations into the computation of network weights and activations. During forward propagation, these operations emulate the clipping and rounding errors associated with mapping FP32 values to INT8, while parameter updates during backpropagation compensate for the resulting quantization error. In this manner, QAT can reduce memory requirements and computational overhead during inference while preserving detection accuracy, making it particularly suitable for deployment on embedded devices and edge-computing platforms [52,53]. The broader development of lightweight intelligent models, including compact vision-language architectures for IoT applications, similarly reflects increasing interest in reducing computational requirements while retaining practical inference capability [54].

Among commonly used model quantization approaches, Post-Training Quantization (PTQ) requires no retraining or only a small calibration dataset, offering a simple implementation pipeline, low computational cost, and high deployment efficiency. However, when the input distribution is complex or the task is highly sensitive to detection accuracy, PTQ may incur substantial performance degradation because quantization errors cannot be compensated during model optimization [55]. By contrast, QAT continuously exposes the model to quantization effects during training or fine-tuning and mitigates the adverse influence of low-bit representations on feature extraction and prediction through parameter optimization. It therefore generally provides more stable performance under low-precision settings such as INT8.

Express-parcel detection involves substantial variation in object size, orientation, appearance, and background conditions, while quantization may further perturb the representation of local textures, object boundaries, and high-level semantic features. To reduce inference cost while preserving detection performance, QAT is applied to the proposed detector through four stages: (1) the trained FP32 model is used for initialization; (2) fake-quantization operations are inserted into key layers to emulate INT8 quantization and dequantization of weights and activations; (3) the quantization-aware model is fine-tuned so that its parameters adapt to quantization error while layer-wise quantization parameters are learned; and (4) the optimized model is converted and exported to INT8 using the learned scale factors and zero points. The complete procedure is summarized in Table 3.

### 2.4. Proposed Model

To address object-scale variation, diverse parcel appearances, stacking and occlusion, boundary ambiguity, and resource constraints in practical deployment, this study develops an improved detector based on YOLOv11s by integrating Selective Feature Aggregation Convolution (SFAConv) and the NeLU-enhanced Global Attention Mechanism (NGAM). The proposed architecture enhances feature representation from two complementary perspectives: lightweight multi-scale feature extraction and deep global semantic modeling, while preserving the efficient inference characteristics of the YOLO framework. The overall architecture is shown in Figure 6.

For lightweight multi-scale feature extraction, selected 3×3 convolutional layers in the Backbone are replaced with SFAConv modules. SFAConv combines depthwise separable convolution, dilated convolution, and adaptive scale selection to capture complementary local textures, object contours, and broader structural patterns under different receptive fields. The scale-selection mechanism dynamically reweights branch responses according to the input features, emphasizing scale-specific information while limiting parameter count and computational complexity. This design improves the representation of parcels with substantial size variation without relying on increased model capacity.

For deep semantic modeling, NGAM is introduced into the deeper Backbone stages to strengthen high-level feature representations. Its channel- and spatial-attention branches jointly model inter-channel dependencies and spatial context, enhancing target-relevant responses while suppressing interference from complex backgrounds, occlusion, and stacking. The NeLU activation further improves nonlinear feature transformation without exponential operations, allowing NGAM to strengthen global semantic modeling with limited additional computational overhead. The original multi-scale feature fusion structure of the YOLOv11s Neck is retained to preserve hierarchical information exchange. Bidirectional top-down and bottom-up feature propagation integrates the spatial detail and localization information of shallow features with the semantic information of deeper features, supporting accurate localization of small parcels while maintaining sufficient context for larger or partially occluded targets.

Overall, SFAConv and NGAM provide complementary improvements within the YOLOv11s framework. SFAConv primarily enhances lightweight multi-scale feature extraction and computational efficiency, whereas NGAM strengthens global contextual modeling in deep features. Their integration improves parcel localization and recognition across varying scales, conditions, and background complexities while maintaining a relatively low model complexity suitable for resource-constrained deployment.

### 2.5. Training Settings and Evaluation Metrics

#### 2.5.1. Model Training Device and Parameters Setups

All model training and evaluation experiments were conducted in a Linux environment using an 8-core CPU, a single NVIDIA B200 GPU, and 32 GB of system memory. For all experiments, the input images were resized to 640 × 640 pixels for both model training and inference, following the standard input configuration of the YOLO framework. During training, the batch size was set to 16, the initial learning rate to 0.001, and AdamW was used for optimization with a weight decay of 0.0005 to regularize model parameters and mitigate overfitting. The maximum number of training epochs was set to 800, with Early Stopping applied using a patience of 50 epochs. Training was terminated when validation performance failed to improve for 50 consecutive epochs, and the model weights corresponding to the best validation performance were retained.

#### 2.5.2. Evaluation Metrics

To evaluate express-parcel detection performance, Precision (*P*), Recall (*R*), $F_{1}$ Score, mean Average Precision (mAP), and GFLOPs were adopted. Precision measures the proportion of correct positive predictions and reflects false-positive control, whereas Recall quantifies the proportion of ground-truth objects successfully detected and reflects missed detections. The $F_{1}$ Score is the harmonic mean of Precision and Recall, providing a balanced measure of detection performance. mAP_50_ denotes mean Average Precision across all classes at an Intersection over Union (IoU) threshold of 0.5, while mAP in this study refers to the average over IoU thresholds from 0.5 to 0.95 with a step of 0.05, providing a stricter assessment of localization performance. GFLOPs was used to quantify computational complexity and support joint evaluation of detection accuracy and efficiency. The mathematical definitions of these metrics are given in Equations (7) and (8).

(7) $$P=\frac{TP}{TP+FP},\;R=\frac{TP}{TP+FN},\;F_{1}=\frac{2\times P\times R}{P+R}$$

(8) $$\mathrm{mAP}=\frac{\sum_{n=1}^{N}\mathrm{AP}\left(n\right)}{N},\;\mathrm{GFLOPs}=\frac{\mathrm{FLOPs}}{10^{9}}$$

Here, TP (True Positive) denotes the number of objects correctly detected by the model; FP (False Positive) denotes the number of incorrect detections; FN (False Negative) denotes the number of ground-truth objects that are not detected; AP represents the Average Precision for a single class; *N* denotes the total number of object classes; and FLOPs represents the total number of floating-point operations performed during a single forward inference pass.

## 3. Experimental Results

### 3.1. Comparative Experiments

To comprehensively evaluate the performance of EPR-YOLO for express-parcel detection, lightweight (n/t) and small-scale (s) variants from the YOLOv8–YOLOv12 and YOLO26 families were selected as comparative models. To broaden the architectural diversity of the comparison beyond the YOLO family, RT-DETR [56,57], a Transformer-based real-time object detector, was additionally included as a representative detector based on a different technical framework. The models were evaluated jointly in terms of detection accuracy, model complexity, and computational cost. All models were trained using the same optimization strategy, and the EPR dataset was partitioned into training, validation, and test sets at a ratio of 7:2:1. Unlike the preliminary 8:2 split used for baseline selection, this partition reserved an independent test set for the final evaluation of model generalization. The test set was kept isolated from model training, validation, and hyperparameter tuning. To reduce the influence of random data partitioning, each model was independently trained and tested using three different random seeds. The final results are reported as the mean and sample standard deviation of each metric, as summarized in Table 4.

Overall, the lightweight models exhibited substantially lower computational complexity and parameter counts (6.1–8.4 GFLOPs and 2.01–3.01 M parameters), but generally underperformed their corresponding small-scale variants in key detection metrics, including Recall, mAP_50_, and $F_{1}$. This indicates that excessive model compression can compromise feature representation capability. By contrast, the small-scale variants generally achieved stronger detection performance, albeit at the cost of increased model complexity, highlighting a clear trade-off between predictive accuracy and computational efficiency. The inclusion of RT-DETR further extends the comparison beyond the YOLO family, providing a cross-framework reference for evaluating the detection performance and computational characteristics of EPR-YOLO.

Compared with the YOLOv11s baseline, EPR-YOLO showed mean increases of 0.857, 0.377, 0.854, 0.022, and 0.574 percentage points in Precision, Recall, mAP_50_, mAP, and $F_{1}$, respectively. Meanwhile, computational complexity decreased from 21.6 to 20.9 GFLOPs, and the parameter count was reduced from 9.43 M to 8.53 M, corresponding to reductions of approximately 3.2% and 9.5%, respectively. However, the numerical improvements in the detection metrics were relatively small compared with the variability observed across the three random experiments. Therefore, these differences should not be interpreted as evidence of statistically significant superiority over YOLOv11s. Rather, the results indicate that EPR-YOLO maintains comparable detection performance while reducing model complexity, with favorable mean values for several detection metrics. Across the three independent experiments, EPR-YOLO exhibited standard deviations of 0.679 and 0.362 percentage points for Precision and mAP_50_, respectively. Taken together, the results suggest that the main advantage of EPR-YOLO lies in achieving a favorable trade-off between detection performance and model efficiency, rather than a large absolute improvement in detection accuracy.

Notably, EPR-YOLO exhibited standard deviations of only 0.679 and 0.362 for Precision and mAP_50_, respectively, across the three independent random experiments. The overall variation was lower than that of most comparative models, indicating favorable training stability and result consistency. Taken together, EPR-YOLO improves detection performance while maintaining relatively low model complexity, achieving a favorable balance among detection accuracy, computational efficiency, and deployment feasibility.

### 3.2. Ablation Study

To quantify the individual contributions and complementary effects of SFAConv and NGAM, YOLOv11s was adopted as the baseline model (X), and three ablation variants were evaluated under identical dataset partitions, optimization strategies, and hyperparameter settings. R_SFAConv retains only NGAM, R_NGAM retains only SFAConv, and EPR-YOLO incorporates both modules. The results are summarized in Table 5.

When only NGAM was retained, R_SFAConv achieved the highest Recall and $F_{1}$ values of 73.047% and 79.605%, representing improvements of 1.913 and 0.899 percentage points over the baseline, respectively. However, Precision and mAP_50_ decreased to 87.514% and 68.761%, while computational complexity increased from 21.6 to 22.3 GFLOPs. These results indicate that NGAM improves target sensitivity and reduces missed detections, but does not independently provide the best balance between detection performance and computational cost.

When only SFAConv was retained, R_NGAM achieved the lowest complexity, with 19.7 GFLOPs and 8.11 M parameters, corresponding to reductions of approximately 8.8% and 14.0% relative to the baseline. It also obtained the highest mAP of 67.713%, suggesting improved localization performance under stricter IoU criteria. However, Recall and $F_{1}$ decreased to 69.672% and 77.662%, indicating that lightweight multi-scale feature extraction alone does not fully preserve target detection capability in complex scenes.

The complete EPR-YOLO model exhibited a different performance balance from either single-module variant. Although its Recall (71.511%) and $F_{1}$ (79.280%) were slightly lower than those of the NGAM-only variant (73.047% and 79.605%, respectively), EPR-YOLO achieved higher Precision and mAP_50_ while reducing GFLOPs from 22.3 to 20.9. This result indicates that the effects of SFAConv and NGAM are not strictly additive across individual detection metrics. In particular, incorporating SFAConv alongside NGAM introduces a trade-off in which part of the Recall advantage associated with NGAM alone is reduced in exchange for improved Precision, mAP_50_, and computational efficiency. Therefore, the functional complementarity of the two modules should be understood in terms of their different contributions to the overall accuracy–efficiency trade-off rather than simultaneous improvement in every metric.

### 3.3. Test Set Evaluation

To further evaluate the generalization performance of the proposed model on independent test data and its ability to retain detection accuracy after quantized deployment, the s variants of the YOLO series, EPR-YOLO, and QAT-EPR-YOLO were compared under a unified test protocol. The evaluation followed the same data-partitioning and repeated-experiment strategy described above: models were trained and tested using three independent random splits, and the mean and sample standard deviation of each metric were reported. Because the test sets were excluded from model training, validation, and hyperparameter tuning, this evaluation provides a more direct assessment of model generalization and deployment robustness.

As shown in Table 6, YOLOv11s, YOLOv12s, YOLO26s, and EPR-YOLO generally outperformed YOLOv8s, YOLOv9s, and YOLOv10s, indicating that the more recent YOLO variants provide stronger feature representation and localization capability for this task. Among them, EPR-YOLO achieved the highest Recall and mAP, reaching 70.730% and 66.160%, respectively, demonstrating advantages in target coverage and localization performance under stricter IoU criteria. Its mAP_50_ reached 70.962%, only 0.023% lower than the 70.985% achieved by YOLOv12s, while its $F_{1}$ score of 78.247% differed by only 0.098% from the highest value of 78.345% obtained by YOLOv12s. These results show that EPR-YOLO does not outperform all competing models on every test set metric. In particular, YOLOv12s achieved marginally higher mAP_50_ and $F_{1}$ values, whereas EPR-YOLO achieved higher Recall and mAP. Given the very small numerical differences among these models, the test results are more appropriately interpreted as demonstrating comparable generalization performance with different metric-specific trade-offs rather than uniform superiority of EPR-YOLO.

A comparison before and after quantization further shows that QAT-EPR-YOLO achieved a Precision of 88.420%, slightly higher than the 88.343% of the original EPR-YOLO. However, Recall, mAP_50_, mAP, and $F_{1}$ decreased by 1.199, 0.263, 0.232, and 0.387 percentage points, respectively, indicating only limited performance degradation after INT8 quantization. The slight increase of 0.077 percentage points in Precision should not be interpreted as a systematic improvement caused by quantization, particularly because its magnitude is small relative to the variation across repeated experiments. This minor change may arise from stochastic training variation and potentially from the regularization effect introduced during quantization-aware fine-tuning. Therefore, the overall effect of QAT is more appropriately characterized as preserving detection performance under reduced numerical precision rather than improving predictive accuracy. Since QAT does not alter the network architecture, the GFLOPs and parameter count remain unchanged, and its lightweight effect is mainly achieved through lower-precision numerical representation. After quantization, the model file size decreased from 19.2 MB to 11.9 MB, corresponding to a reduction of approximately 38.0%, thereby reducing storage requirements and model-loading overhead. Overall, QAT-EPR-YOLO largely preserves the detection capability of the FP32 model while reducing storage cost, supporting its feasibility for lightweight deployment.

### 3.4. Web-Based Application

To further assess the engineering applicability and practical deployment potential of the proposed intelligent express-parcel detection model, a Web-based intelligent parcel detection application was designed and implemented after model development, performance optimization, and lightweight quantization. The platform uses the trained EPR-YOLO model as its core inference engine and integrates a front-end visualization interface with a back-end inference service to support image uploading, object detection, result visualization, batch processing, and runtime status monitoring. Through this system, users can perform express-parcel detection tasks through an intuitive and accessible interface, providing an application-level evaluation of the deployment feasibility and practical value of the proposed model in intelligent logistics scenarios.

The system adopts a lightweight front-end/back-end collaborative architecture, as illustrated in Figure 7. The front end is implemented as a Single Page Application (SPA) using native HTML, CSS, and JavaScript, and is responsible for user interaction, image uploading, detection request submission, result rendering, and batch-task management. This design does not rely on additional front-end build tools such as Webpack or Vite, allowing static pages and resources to be directly hosted by the FastAPI back end. Consequently, deployment complexity is reduced and portability across different runtime environments is improved. The back end is implemented with FastAPI and provides model loading, API handling, image preprocessing, object detection inference, and structured result generation. The front end asynchronously invokes three core endpoints through the Fetch API, namely /api/health, /api/detect, and /api/detect/batch, which are responsible for model-status queries, single-image detection, and batch detection, respectively. After an image is uploaded, the back end performs image parsing, preprocessing, and model inference, and returns the annotated image, predicted classes, confidence scores, bounding-box coordinates, processing time, and class distribution in JSON format. The front end then parses and visualizes these results, completing the full workflow from image input to detection output.

To support online demonstration and practical application of express-parcel detection, the platform is organized into four core modules: system overview, single-image detection, batch detection, and runtime monitoring. The system overview module displays model loading status, computing device, confidence threshold, and class information, allowing users to quickly verify the operational state of the platform. The single-image detection module supports image upload, inference, and result visualization, while simultaneously reporting the number of detected objects, average confidence, inference time, class statistics, and detailed detection results, as illustrated in Figure 8. The batch detection module is designed for continuous image-processing scenarios and supports unified upload, batch inference, and result aggregation for multiple images. The runtime monitoring module records the number of processed images, total detected objects, average response time, and key operation logs, thereby improving the observability of system execution.

The main interface adopts semantic HTML, CSS Grid, Flexbox, and media queries to provide responsive layout adaptation, ensuring clear information organization and consistent interaction workflows on both desktop and smaller-screen devices. Overall, the Web platform not only visualizes express-parcel detection results but also summarizes parcel categories, quantities, and distributions. These functions provide data support for logistics sorting, resource scheduling, and anomaly monitoring, while offering an application prototype for subsequent system integration and practical deployment.

## 4. Discussion

### 4.1. Advantages

The proposed intelligent express-parcel detection framework establishes a complete technical pipeline spanning dataset construction, model optimization, lightweight deployment, and application-level validation. Its contribution is not limited to improvements in individual detection metrics, but lies in the coordinated consideration of data quality, detection performance, computational complexity, and deployment feasibility. To address the fragmented sources, inconsistent label taxonomies, and high annotation cost of existing express-parcel datasets, this study integrates open-source datasets, Internet-sourced images, and field-acquired images, and combines unified re-annotation with an Active Learning-based pseudo-labeling strategy. This approach expands data diversity and scenario coverage while maintaining annotation consistency through unified class definitions and manual correction, thereby reducing dependence on fully manual annotation during dataset expansion. It should be noted that the architectural contribution of EPR-YOLO does not lie in proposing entirely new primitive operations. SFAConv is adapted from an existing lightweight multi-scale convolution design, while NGAM combines the established GAM structure with NeLU-based nonlinear transformation. The contribution of the present work is instead the task-oriented integration and placement of these components within YOLOv11s for express-parcel detection, together with their joint evaluation under a unified lightweight detection framework. The ablation results further show that these components introduce different metric-specific effects rather than uniformly additive improvements, emphasizing that the proposed architecture should be viewed as an application-oriented optimization of the accuracy–complexity trade-off.

At the model level, the proposed EPR-YOLO is built on YOLOv11s and improves multi-scale feature extraction and deep semantic modeling through SFAConv and NGAM, respectively. SFAConv reduces model complexity through a lightweight multi-scale convolutional structure, whereas NGAM enhances global feature modeling through coordinated channel and spatial attention. These two modules therefore provide functionally complementary improvements. Comparative and ablation experiments further demonstrate that EPR-YOLO improves multiple detection metrics while simultaneously reducing parameter count and computational cost, indicating that its performance gains are not achieved through increased model scale, but primarily through more efficient feature extraction and representation. As a result, the proposed architecture provides a more favorable trade-off between detection performance and computational efficiency.

For lightweight deployment, Quantization-Aware Training (QAT) is further applied to convert EPR-YOLO to INT8 precision. Independent test results show that quantization introduces only limited performance degradation, with the major detection metrics remaining close to those of the FP32 model. This indicates that the proposed model is well suited to low-precision representation. Unlike evaluations that characterize model lightweightness solely through parameter count or FLOPs, QAT further verifies the feasibility of model compression and low-precision inference from the perspective of numerical representation, providing experimental support for deployment on platforms with constrained computational and storage resources.

On this basis, a FastAPI-based Web intelligent detection system is developed to integrate the proposed model into a practical inference workflow, supporting single-image and batch detection, result visualization, and structured output. The system is not intended to replace hardware-level validation on real logistics terminals, but rather to serve as a prototype for evaluating model integration and online inference capability. Consequently, the overall study forms a coherent framework linking dataset construction, model design, quantization optimization, and application implementation. This not only demonstrates the experimental advantages of the proposed method, but also provides a technical foundation for subsequent deployment and validation on practical logistics devices.

### 4.2. Challenges and Future Directions

Despite the construction of the EPR dataset through multi-source data integration and Active Learning-based pseudo-label iteration, its current scale and scenario coverage remain limited. The existing samples are primarily concentrated on common express-parcel categories and representative logistics environments, while challenging conditions such as severe occlusion, dense multi-object stacking, transparent or special-material packaging, and extreme illumination are still underrepresented. Future work should therefore expand data acquisition across different logistics companies, warehouse environments, and transportation stages, while incorporating continual learning or active learning strategies for dynamic dataset updating [10,11]. Such extensions would improve the representativeness of the data distribution and reduce model dependence on specific acquisition environments.

In addition, the present study primarily evaluates the EPR dataset and detection results at the overall dataset level, without conducting a detailed class-wise analysis of sample distribution and detection performance. Future work will further investigate category-level data distribution and class-wise detection metrics to assess the potential effects of class imbalance and identify category-specific limitations of the detector. Representative failure cases were also not systematically visualized in the present study. Potential detection errors may arise from severe occlusion, overlapping parcels, large variations in object scale and appearance, and visually similar parcel categories, which may lead to missed detections or incorrect classifications. These challenging cases will be investigated more systematically in future work.

At the model level, EPR-YOLO has thus far been trained and validated primarily on the EPR dataset, and its cross-dataset and cross-scenario generalization capability requires more systematic evaluation. In particular, substantial changes in imaging devices, background distributions, or target appearances may introduce domain shifts that degrade detection performance [58]. Future research could therefore conduct cross-domain evaluations and incorporate domain adaptation, domain generalization, self-supervised learning, or open-vocabulary detection to improve adaptability to unseen environments and emerging parcel categories [59,60,61]. The practical gains of these strategies should also be evaluated jointly in terms of detection accuracy and model complexity [21,62,63].

Across other data-intensive application domains, recent studies have also emphasized the integration of heterogeneous data, computational modeling, and intelligent analysis for supporting complex system assessment and decision-making [64,65,66]. Although these studies address application contexts different from logistics vision, they illustrate the broader development of data-driven methodologies for analyzing heterogeneous operating conditions and complex system interactions.

More broadly, data-driven learning has demonstrated strong adaptability across heterogeneous scientific and engineering domains, including environmental risk modeling, intelligent sensing, biomedical imaging, and three-dimensional reconstruction [67,68,69,70]. These developments provide broader methodological references for extracting task-relevant representations from complex and heterogeneous observations. For express-parcel detection, future work should specifically examine whether representations learned from the current EPR dataset remain reliable under changes in imaging devices, warehouse layouts, illumination conditions, and parcel appearances.

Related research on image-based phenotyping, three-dimensional reconstruction, and heterogeneous sensing has explored high-throughput imaging, neural radiance fields, 3D deep learning, point-cloud segmentation, Gaussian splatting, video-based tracking, internal-quality assessment, neural architecture search, probabilistic transfer learning, and atmospheric-parameter diagnosis [71,72,73,74,75,76,77,78,79,80]. These studies provide broader methodological context for learning robust representations from complex and heterogeneous observations, which may inform future investigation of parcel perception under larger domain shifts and richer sensing conditions.

Future logistics perception systems may also benefit from multimodal and multi-sensor information integration. Multimodal learning has demonstrated the capability to combine heterogeneous visual, semantic, and contextual information in applications involving video understanding and cross-modal prediction [81,82], while multi-sensor information fusion provides a complementary strategy for integrating measurements from heterogeneous sensing sources [83]. Extending parcel perception beyond individual RGB images could therefore enable the integration of depth information, barcode or OCR recognition, temporal tracking, and other sensing modalities in more complex logistics environments.

Recent studies on vision-language adaptation and reasoning have further explored parameter-efficient model tuning, explicit visual reasoning, multimodal reasoning, text-to-structure reasoning, and view-aligned multimodal learning [84,85,86,87,88]. These directions may provide useful methodological references if future parcel-perception systems integrate visual detection with semantic reasoning or higher-level task understanding.

In addition to generalization across logistics environments, the reliability and trustworthiness of learned models warrant further consideration as intelligent perception systems become increasingly interconnected. Recent research on the robustness and security of learned models provides useful methodological perspectives for evaluating model reliability beyond conventional predictive metrics [89].

Regarding deployment, QAT experiments demonstrate that EPR-YOLO can largely preserve its detection performance under INT8 quantization while reducing model storage requirements, and Web-based inference has also been successfully validated through the developed FastAPI application. However, due to limitations in the availability of appropriate edge hardware and experimental conditions, the quantized model has not yet been benchmarked on actual resource-constrained edge devices. Therefore, the current results demonstrate the potential for lightweight deployment rather than validated edge-device deployment performance. Differences among GPUs, NPUs, and embedded platforms in operator support, memory bandwidth, and inference frameworks may substantially affect practical deployment behavior. Accordingly, device-level latency, throughput, memory consumption, and energy efficiency of the quantized model require further evaluation [90]. Future work will extend the current Web-based validation by conducting on-device deployment experiments on representative logistics terminals and edge AI platforms, thereby providing a more comprehensive assessment of real-time performance and engineering applicability under resource-constrained conditions [91].

Beyond quantization, recent studies on workload synthesis, performance-aware query generation, diffusion-model acceleration, low-rank inference, shared decode-time representations, and scalable optimization have investigated complementary routes to computational efficiency [92,93,94,95,96,97,98,99]. These developments provide broader computational context for future optimization of online parcel-inference services when latency, throughput, memory consumption, and computational cost must be considered jointly.

Computational optimization represents another relevant direction for future system development. Iterative numerical optimization and constraint-aware multi-objective optimization have been investigated in complex engineering problems involving multiple interacting design requirements [100,101]. Similar optimization principles could be explored in future work to jointly balance parcel-detection performance, computational complexity, inference latency, and resource consumption.

In addition, the current Web system is primarily designed to validate parcel detection and result visualization, whereas practical intelligent logistics workflows typically involve multiple interconnected functions, including object tracking, barcode or OCR recognition, parcel-condition assessment, robotic grasping, and sorting decision-making [102,103,104,105].

As the current Web prototype evolves toward interconnected logistics information systems, secure data exchange and system-level information protection will also become relevant considerations. Existing studies on privacy-preserving distributed transactions, authenticated group communication, and information security in smart manufacturing provide methodological references for the design of secure interconnected intelligent systems [106,107,108]. Performance modeling of distributed computing and communication processes may additionally provide useful tools for analyzing service efficiency as such systems scale to multiple devices or concurrent inference requests [109].

Future work could extend the present framework by integrating multi-object tracking, multimodal recognition, and robotic execution modules to establish a complete closed-loop pipeline from visual perception to task decision and execution. Compared with evaluating a standalone detector, such system-level integration would provide a more realistic assessment of continuous operation capability and practical deployment value in real logistics workflows.

Probabilistic models such as Bayesian networks have also been employed to characterize relationships among multiple interacting factors in complex operational systems [110]. Such probabilistic reasoning could provide a complementary direction for future logistics decision modules when visual detection outputs need to be integrated with operational-state information.

Beyond visual perception, closed-loop logistics automation also requires effective interaction among perception, manipulation, and control. Recent developments in adaptive and fault-tolerant nonlinear control provide methodological references for maintaining prescribed system behavior under disturbances, nonlinear dynamics, and actuator uncertainties [111,112,113], while advances in soft robotic grasping demonstrate increasingly adaptable manipulation mechanisms for objects with diverse geometries [114]. These developments provide useful directions for the future integration of EPR-YOLO with robotic parcel grasping and automated sorting systems.

## 5. Conclusions

This study proposes a lightweight express-parcel detection framework for complex logistics environments. The Express Parcel Recognition (EPR) dataset is first constructed through multi-source data acquisition, unified re-annotation, and Active Learning-based pseudo-labeling to reduce manual annotation effort while maintaining label consistency. Using YOLOv11s as the baseline, SFAConv and NGAM are incorporated to develop EPR-YOLO, enhancing multi-scale feature extraction and global semantic modeling with reduced computational complexity. Quantization-Aware Training is further applied for INT8 deployment. Comparative, ablation, and independent test set experiments show that EPR-YOLO achieves stable detection performance with low model complexity, while the quantized model largely retains the accuracy of its floating-point counterpart. A FastAPI-based Web application is also developed to support single-image and batch detection, result visualization, and online inference, validating the feasibility of system integration and practical deployment. Overall, the proposed framework integrates dataset construction, lightweight model optimization, quantization, and application deployment for resource-constrained express-parcel detection.

## Figures and Tables

**Figure 1:**
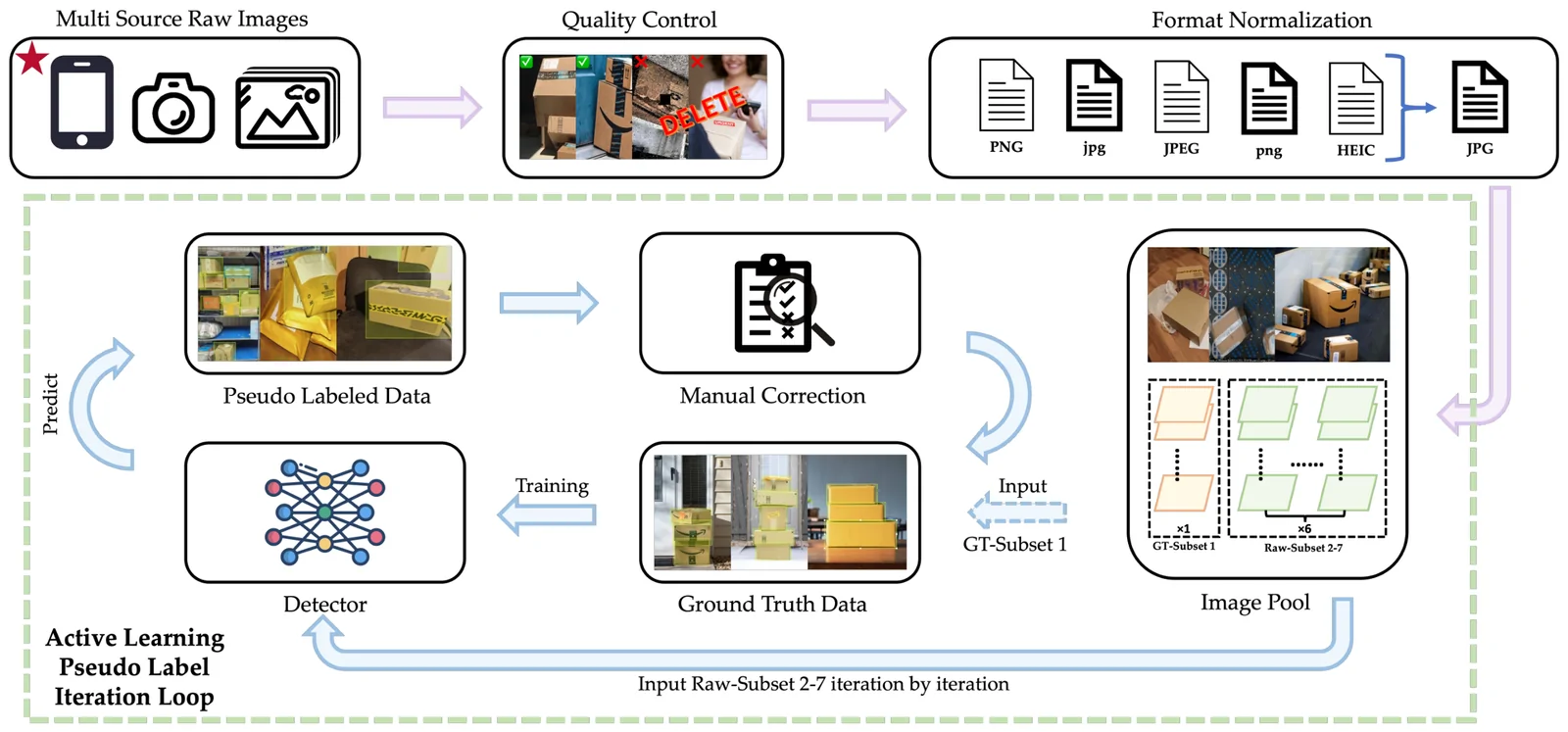
Workflow of active learning pseudo-label iteration dataset construction. Note: Flows in the iteration loops are highlighted by blue arrows; blue dashed arrow represents that the operation is performed only once; the red star marks the start of the entire pipeline.

**Figure 2:**
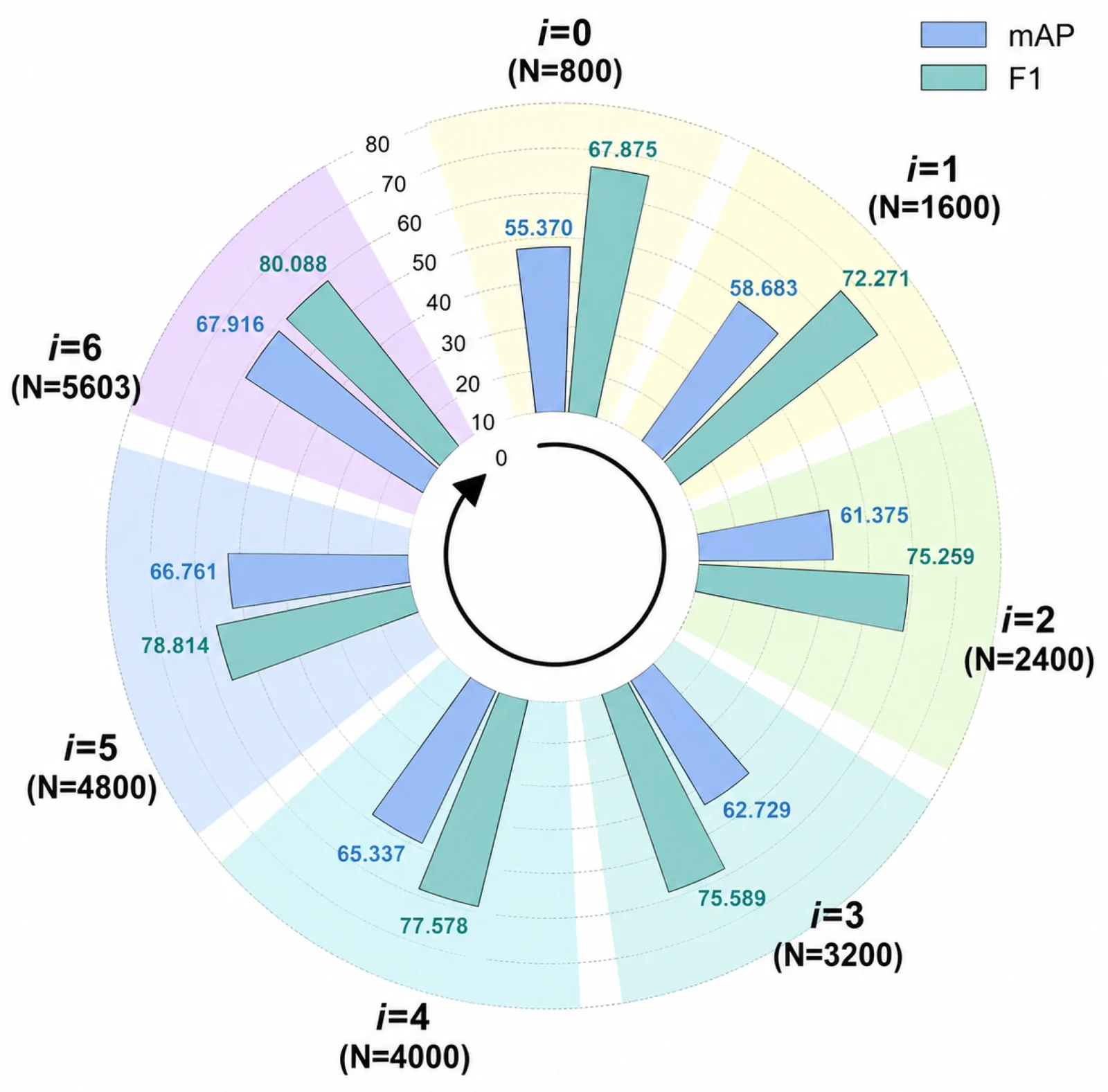
Evolution of mAP and $F_{1}$ during the seven-round active-learning-based pseudo-label iteration process. Here, *i* denotes the iteration index (i=0,1,2,3,4,5,6), and *N* represents the sample size of the labeled dataset at each iteration.

**Figure 3:**
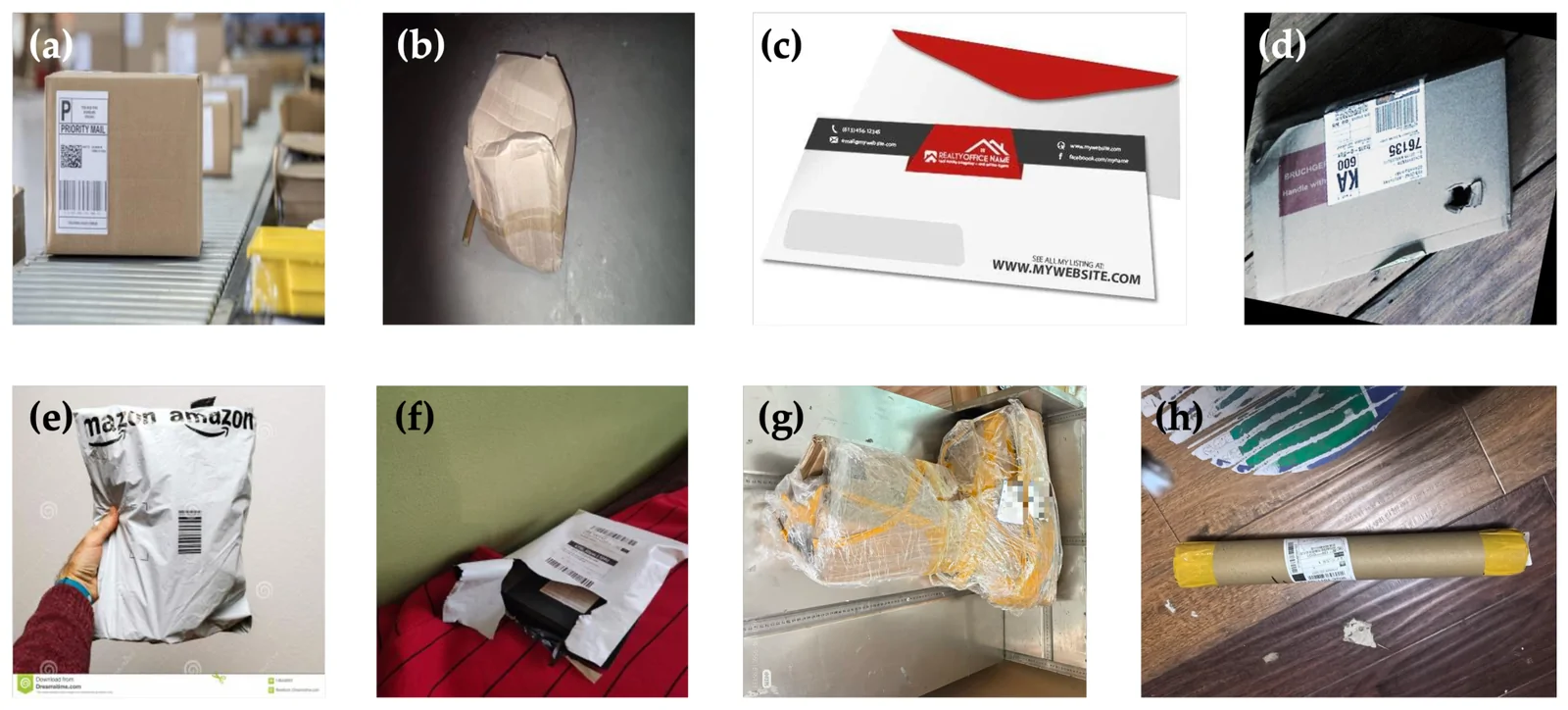
Representative images of EPR dataset samples. **Note:** (**a**) Box; (**b**) damaged box; (**c**) envelope; (**d**) damaged envelope; (**e**) poly mailer; (**f**) damaged poly mailer; (**g**) irregular; (**h**) tube.

**Figure 4:**
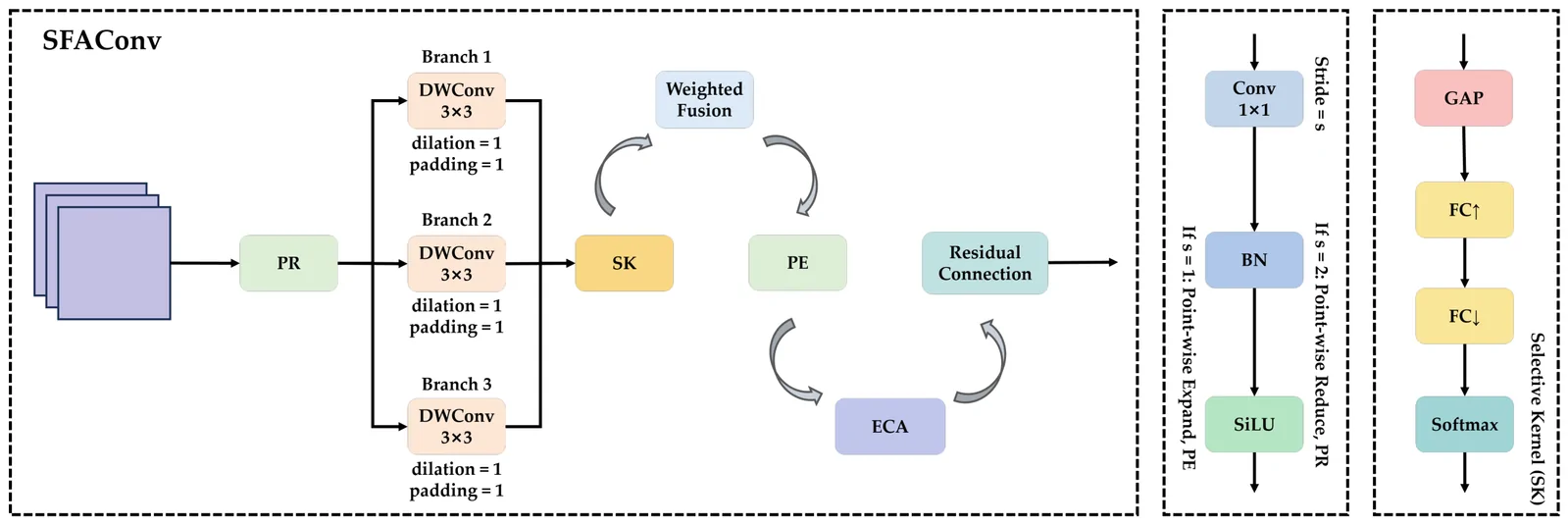
Schematic diagram of the Selective Multi-Scale Feature Aggregation Convolution (SFAConv) structure.

**Figure 5:**
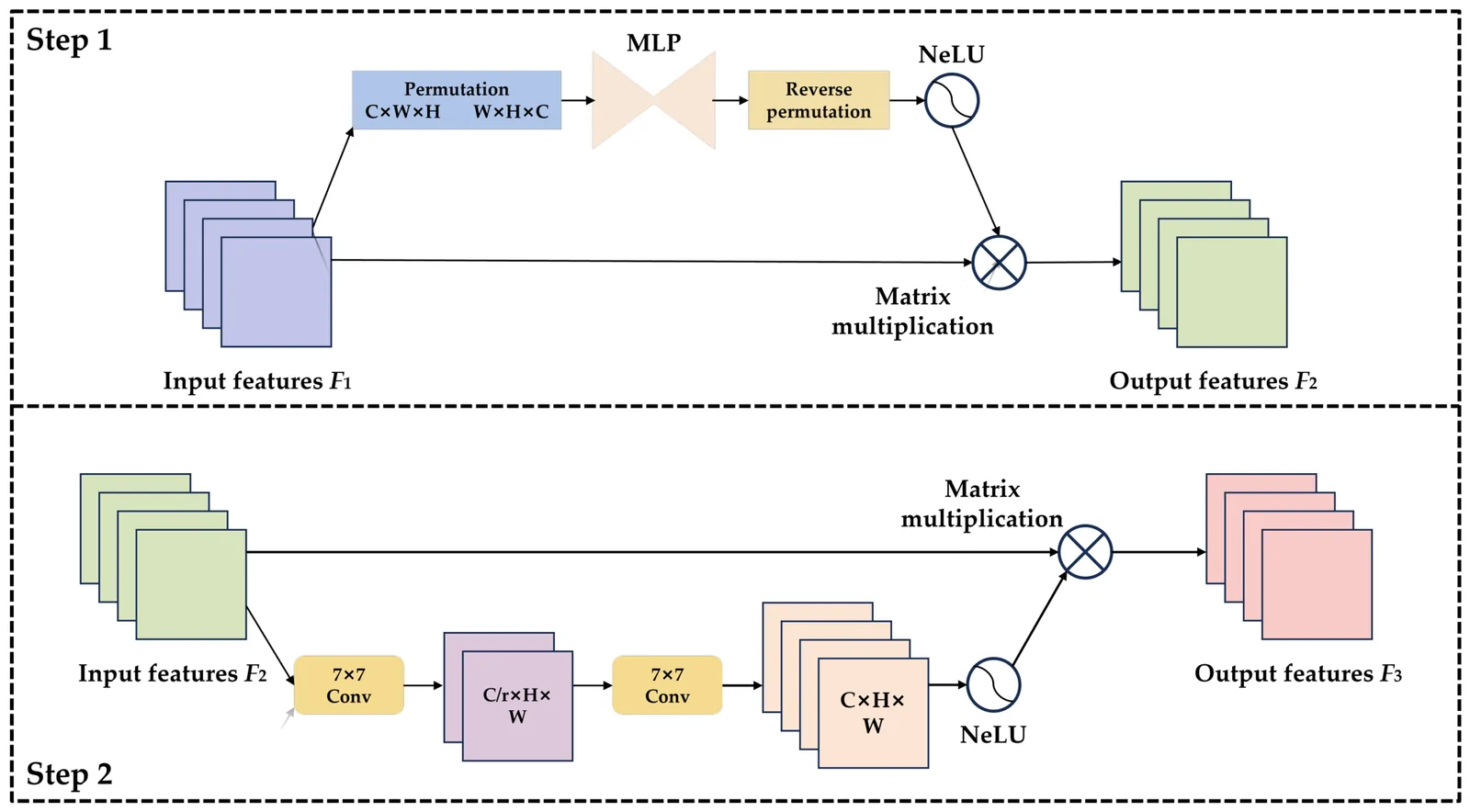
Schematic diagram of the NGAM attention module structure.

**Figure 6:**
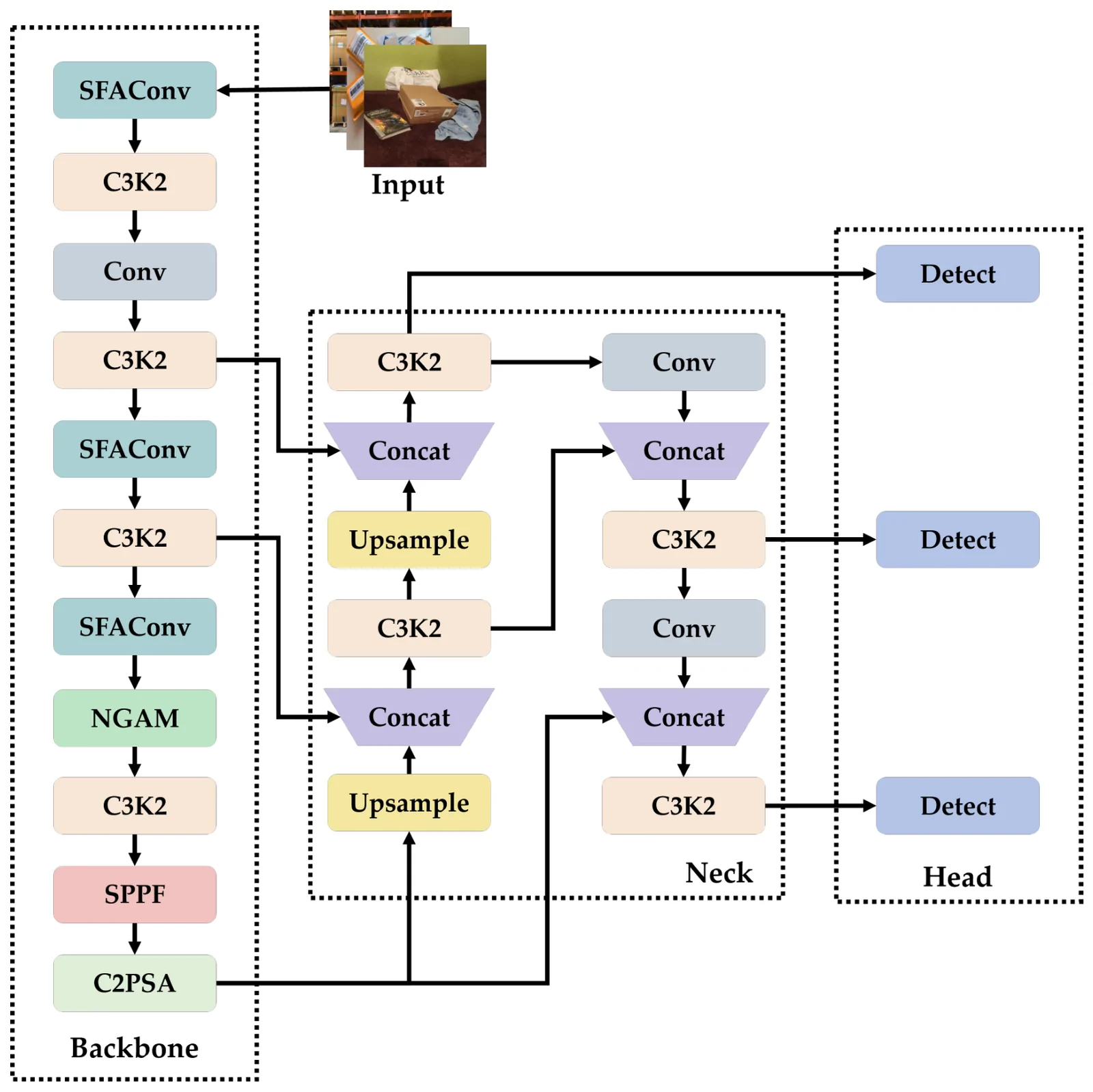
Schematic diagram of the EPR-YOLO architecture.

**Figure 7:**
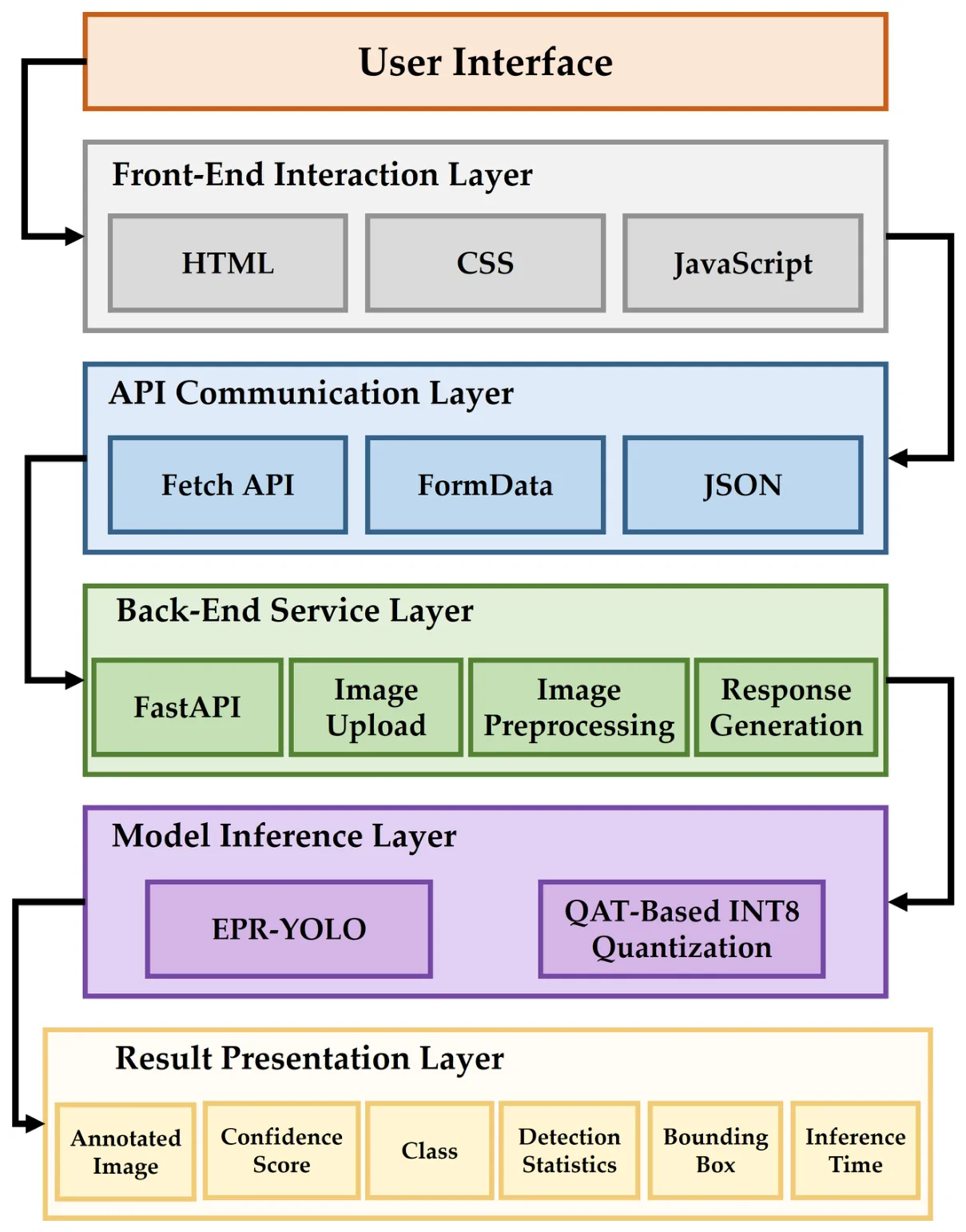
Overall architecture of the Web-based EPR-YOLO parcel detection system.

**Figure 8:**
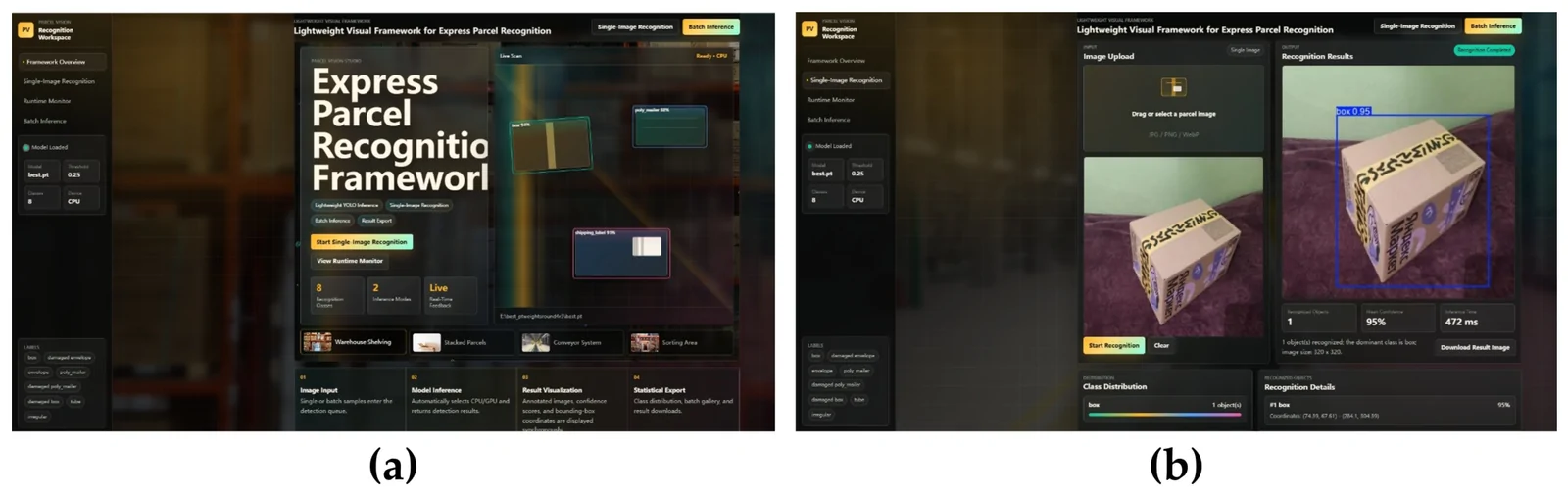
Web-based parcel detection application: (**a**) main interface; (**b**) visualization of parcel detection results.

**Table 1:** Baseline model selection results during the complete dataset construction stage.

Model	*P* (%)	*R* (%)	mAP_50_ (%)	mAP (%)	$F_{1}$ (%)
YOLOv8n	80.803±11.570	59.618±4.933	65.799±4.565	53.574±4.256	68.047±1.838
YOLOv8s	75.842±8.124	59.789±7.727	70.322±3.307	54.497±6.718	66.515±5.654
YOLOv9t	76.027±6.444	54.342±3.545	60.997±5.578	50.709±6.751	63.178±1.162
YOLOv9s	75.447±6.851	59.846±4.698	65.604±4.969	54.707±4.279	66.442±1.063
YOLOv10n	67.800±16.026	51.310±4.015	56.974±7.553	44.975±7.374	57.953±7.981
YOLOv10s	76.307±6.960	54.934±2.136	63.278±2.067	54.035±1.607	63.705±1.170
YOLOv11n	74.656±5.048	58.696±2.666	68.821±5.400	51.578±3.099	65.642±2.408
YOLOv11s	80.071±3.007	59.033±3.047	66.501±3.490	55.370±3.185	67.875±0.958
YOLOv12n	70.117±6.267	62.752±7.759	64.583±5.092	52.100±5.526	65.852±3.525
YOLOv12s	75.897±9.136	58.928±3.320	64.555±4.569	51.798±1.248	66.027±2.564
YOLO26n	73.691±8.220	58.715±3.823	61.801±2.393	49.648±3.607	65.023±1.568
YOLO26s	83.078±5.978	53.842±6.145	63.913±4.028	51.820±2.858	64.999±2.984

Note: The dataset was randomly split three times, and the mean and sample standard deviation of each metric are reported.

**Table 2:** Baseline model selection results.

Model	*P* (%)	*R* (%)	mAP_50_ (%)	mAP (%)	$F_{1}$ (%)	GFLOPs	Params (M)
YOLOv8n	86.615±0.838	71.091±0.064	70.241±0.134	63.985±1.583	78.088±0.378	8.2	3.01
YOLOv8s	85.307±1.067	70.188±0.871	71.318±0.802	65.354±1.374	77.005±0.286	28.6	11.14
YOLOv9t	82.796±1.735	69.004±0.917	69.078±1.066	62.341±2.152	75.273±1.261	7.8	2.01
YOLOv9s	84.105±1.507	70.716±0.550	70.008±1.141	63.826±1.093	76.829±0.908	27.4	7.29
YOLOv10n	86.052±1.606	71.858±0.273	71.454±0.523	66.761±1.553	78.313±0.664	8.4	2.71
YOLOv10s	86.990±1.991	72.633±0.521	71.720±1.031	67.627±1.584	79.162±1.130	24.8	8.07
YOLOv11n	87.899±1.318	71.568±0.507	71.173±1.721	66.892±2.053	78.893±0.525	6.4	2.59
YOLOv11s	88.597±0.577	73.072±0.606	72.454±1.192	67.916±1.694	80.088±0.449	21.6	9.43
YOLOv12n	88.122±1.985	71.553±1.244	71.236±0.876	67.088±1.943	78.965±0.983	6.5	2.57
YOLOv12s	88.066±1.031	72.350±0.914	72.158±0.968	67.294±1.214	79.430±0.138	21.5	9.25
YOLO26n	89.059±1.293	68.854±1.808	69.865±1.582	66.075±0.523	77.643±0.646	6.1	2.57
YOLO26s	88.758±1.068	70.671±0.657	70.846±0.633	66.852±0.857	78.683±0.130	22.8	10.01

Note: The dataset was randomly divided three times, and the mean and sample standard deviation of each metric are reported.

**Table 3:** Pseudo-code of the quantization-aware training procedure.

**Input:** Trained FP32 model $M_{\mathrm{fp32}}$, training dataset $D_{\mathrm{train}}$, validation dataset $D_{\mathrm{val}}$ **Output:** INT8 model $M_{\mathrm{int8}}$	
**1:**	Insert fake-quantization operators into $M_{\mathrm{fp32}}$.
**2:**	Construct the quantization-aware model $M_{\mathrm{qat}}$.
**3:**	Initialize the best validation score $S_{\mathrm{best}}$.
**4:**	**while** training is not converged **do**
**5:**	Train $M_{\mathrm{qat}}$ using $D_{\mathrm{train}}$.
**6:**	Evaluate the current model on $D_{\mathrm{val}}$ and obtain $S_{\mathrm{cur}}$.
**7:**	**if** $S_{\mathrm{cur}}>S_{\mathrm{best}}$ **then**
**8:**	Save the current model and update $S_{\mathrm{best}}$.
**9:**	**end if**
**10:**	**end while**
**11:**	Export quantization parameters.
**12:**	Convert $M_{\mathrm{qat}}$ into the INT8 model $M_{\mathrm{int8}}$.

**Table 4:** Comparative performance of EPR-YOLO, representative lightweight and small YOLO-series models, and Transformer-based object detection models on the EPR dataset.

Model	*P* (%)	*R* (%)	mAP_50_ (%)	mAP (%)	$F_{1}$ (%)	GFLOPs	Params (M)
YOLOv8n	83.953±0.910	68.763±0.879	68.959±0.826	63.048±1.208	75.602±0.851	8.2	3.01
YOLOv8s	85.649±0.918	70.226±0.617	70.499±0.474	63.705±2.303	77.174±0.747	28.6	11.14
YOLOv9t	81.764±2.044	67.729±0.466	68.056±1.151	62.103±1.888	74.079±0.869	7.8	2.01
YOLOv9s	83.895±2.511	69.535±0.939	69.587±1.100	63.717±1.918	76.033±1.280	27.4	7.29
YOLOv10n	82.806±0.965	68.967±1.996	68.935±0.964	63.945±0.983	75.240±1.163	8.4	2.71
YOLOv10s	85.324±1.849	70.729±1.459	70.678±0.321	65.104±0.871	77.324±0.464	24.8	8.07
YOLOv11n	86.360±0.901	69.933±1.127	69.391±0.866	65.082±2.011	77.276±0.343	6.4	2.59
YOLOv11s	88.101±0.992	71.134±0.981	70.800±0.719	66.818±1.857	78.706±0.390	21.6	9.43
YOLOv12n	86.764±1.752	69.036±1.145	69.332±0.521	64.945±1.805	76.876±0.377	6.5	2.57
YOLOv12s	88.583±1.505	70.790±0.792	70.548±1.047	66.250±1.580	78.683±0.347	21.5	9.25
YOLO26n	84.457±0.819	69.206±1.286	69.163±0.888	64.825±1.563	76.071±1.060	6.1	2.57
YOLO26s	86.318±0.646	70.891±0.717	70.852±1.173	66.303±1.260	77.846±0.690	22.8	10.01
RT-DETR	83.361±0.678	70.211±0.565	69.090±0.883	62.722±1.391	76.220±0.058	130.5	42.76
EPR-YOLO	88.958±0.679	71.511±0.942	71.654±0.362	66.840±1.697	79.280±0.407	20.9	8.53

Note: The dataset was randomly divided three times, and the mean and sample standard deviation of each metric are reported.

**Table 5:** Ablation study results.

Model	*P* (%)	*R* (%)	mAP_50_ (%)	mAP (%)	$F_{1}$ (%)	GFLOPs	Params (M)
X	88.101±0.992	71.134±0.981	70.800±0.719	66.818±1.857	78.706±0.390	21.6	9.43
R_SFAConv	87.514±0.797	73.047±1.377	68.761±0.841	66.731±1.698	79.605±0.870	22.3	8.96
R_NGAM	87.723±0.753	69.672±0.709	70.078±0.317	67.713±1.590	77.662±0.520	19.7	8.11
EPR-YOLO	88.958±0.679	71.511±0.942	71.654±0.362	66.840±1.697	79.280±0.407	20.9	8.53

Note: The dataset was randomly divided three times, and the mean and sample standard deviation of each metric are reported.

**Table 6:** Test set validation results.

Model	*P* (%)	*R* (%)	mAP_50_ (%)	mAP (%)	$F_{1}$ (%)
YOLOv8s	84.807±1.827	69.734±0.954	69.759±0.977	63.882±1.515	76.530±1.142
YOLOv9s	83.685±2.627	69.379±0.813	69.366±1.061	63.526±1.557	75.179±1.958
YOLOv10s	84.573±2.010	69.572±0.879	69.547±1.013	63.665±1.723	76.327±0.960
YOLOv11s	87.406±0.247	70.337±1.032	70.355±1.012	65.680±1.568	77.945±0.694
YOLOv12s	88.124±1.341	70.523±0.976	70.985±0.282	66.044±1.595	78.345±0.254
YOLO26s	86.167±1.308	70.526±1.475	70.419±0.444	66.064±1.633	77.545±0.721
EPR-YOLO	88.343±0.643	70.730±0.856	70.962±0.575	66.160±1.930	78.247±0.844
QAT-EPR-YOLO	88.420±0.489	69.531±1.377	70.699±0.546	65.928±1.918	77.860±0.957

Note: The dataset was randomly divided three times, and the mean and sample standard deviation of each metric are reported.

## Data Availability

The raw data supporting the conclusions of this article will be made available by the authors on request. The EPR dataset generated and used in this study is openly available on Kaggle at https://www.kaggle.com/datasets/ongkingu1/express-parcel-recognition-epr-dataset, accessed on 1 September 2026. The project repository and implementation resources are available on GitHub at https://github.com/AnqiWuCW/EPR-YOLO, accessed on 1 September 2026.
